# Characterization of Human Pseudogene-Derived Non-Coding RNAs for Functional Potential

**DOI:** 10.1371/journal.pone.0093972

**Published:** 2014-04-03

**Authors:** Xingyi Guo, Mingyan Lin, Shira Rockowitz, Herbert M. Lachman, Deyou Zheng

**Affiliations:** 1 The Saul R. Korey Department of Neurology, Albert Einstein College of Medicine, New York, New York, United States of America; 2 Department of Genetics, Albert Einstein College of Medicine, New York, New York, United States of America; 3 Department of Neuroscience, Albert Einstein College of Medicine, New York, New York, United States of America; 4 Department of Psychiatry and Behavioral Sciences, Albert Einstein College of Medicine, New York, New York, United States of America; University of California, Los Angeles, United States of America

## Abstract

Thousands of pseudogenes exist in the human genome and many are transcribed, but their functional potential remains elusive and understudied. To explore these issues systematically, we first developed a computational pipeline to identify transcribed pseudogenes from RNA-Seq data. Applying the pipeline to datasets from 16 distinct normal human tissues identified ∼3,000 pseudogenes that could produce non-coding RNAs in a manner of low abundance but high tissue specificity under normal physiological conditions. Cross-tissue comparison revealed that the transcriptional profiles of pseudogenes and their parent genes showed mostly positive correlations, suggesting that pseudogene transcription could have a positive effect on the expression of their parent genes, perhaps by functioning as competing endogenous RNAs (ceRNAs), as previously suggested and demonstrated with the *PTEN* pseudogene, *PTENP1*. Our analysis of the ENCODE project data also found many transcriptionally active pseudogenes in the GM12878 and K562 cell lines; moreover, it showed that many human pseudogenes produced small RNAs (sRNAs) and some pseudogene-derived sRNAs, especially those from antisense strands, exhibited evidence of interfering with gene expression. Further integrated analysis of transcriptomics and epigenomics data, however, demonstrated that trimethylation of histone 3 at lysine 9 (H3K9me3), a posttranslational modification typically associated with gene repression and heterochromatin, was enriched at many transcribed pseudogenes in a transcription-level dependent manner in the two cell lines. The H3K9me3 enrichment was more prominent in pseudogenes that produced sRNAs at pseudogene loci and their adjacent regions, an observation further supported by the co-enrichment of SETDB1 (a H3K9 methyltransferase), suggesting that pseudogene sRNAs may have a role in regional chromatin repression. Taken together, our comprehensive and systematic characterization of pseudogene transcription uncovers a complex picture of how pseudogene ncRNAs could influence gene and pseudogene expression, at both epigenetic and post-transcriptional levels.

## Introduction

Pseudogenes are genomic sequences with high sequence similarity to functional genes but have been presumed to be “non-functional” [1]–[3]. By definition, pseudogenes derived from protein-coding genes have lost their protein-coding capacity due to deleterious disruptions (e.g., premature stop codons or frame shift mutations) in their hypothetical open reading frames. Based on distinct generation mechanisms, pseudogenes are separated into processed pseudogenes (generated by retrotransposition) and duplicated pseudogenes (from gene duplication). This separation is primarily based on examination of sequence features, with the lack of introns as strong evidence for retrotransposition, whereas older pseudogenes with extensive structure degeneration are sometimes classified as pseudogene fragments due to ambiguity. The functional gene with the “highest” sequence similarity to a pseudogene is often operationally referred as its parental gene, which is also used in the current study.

Thousands of pseudogenes are found in the human genome; some of them have been suggested to have critical regulatory functions [4]–[7]. Historically, pseudogenes are considered to be mostly transcriptionally inactive because they are presumably lacking either a functional promoter or auxiliary regulatory elements. However, recent studies have found that a substantial portion of pseudogenes can actually be transcribed to stable RNAs [8]–[12]. Furthermore, accumulating lines of evidence suggest that pseudogenes, *via* their non-coding RNA (ncRNA) products, may play regulatory roles in modulating the expression of their parental genes, as well as non-parental genes [1], [3], [13]–[21]. For example, short interfering RNAs (siRNAs) derived from pseudogenes, through their complementary interactions with mRNAs of the parental genes, were found to down regulate parental gene expression in mouse oocytes by a Dicer-dependent RNAi process [22], [23]. Our recent analysis of millions of small RNAs from multiple rice tissues also supports the idea that high eukaryotic pseudogenes can produce endogenous siRNAs (endo-siRNAs) that are mostly tissue and development-stage specific [24]. Moreover, many of those pseudogene-derived endo-siRNAs share similar features with plant repeat-associated siRNAs that can mediate RNA-directed DNA methylation and heterochromatin formation [24]. Whether mammalian pseudogenes can play a similar role in modulating epigenetic repression at pseudogene loci (i.e., *cis*-effect) has not yet been investigated, although *trans*-effects have been suggested. For example, the *Oct4* pseudogene ncRNA was shown to direct epigenetic remodeling complexes to the *Oct4* parent gene [25].

Pseudogene transcripts functioning by other mechanisms have also been reported [8], [14], [19], [20], [26]–[29], including acting as antisense transcripts [25], [30]. *PTENP1,* a pseudogene derived from the tumor suppressor gene *PTEN*, was first shown to act as a competitive decoy for several miRNAs that target PTEN mRNA, thus stabilize expression of its parental *PTEN* gene [28]. The recent discovery of antisense ncRNAs from *PTENP1* and their role in regulating *PTEN* [31], furthermore, indicates that functional interaction between pseudogenes and their parents can be complex and multilayered. Given the wide range of biological functions potentially carried out by ncRNAs [32]–[34], and the high sequence similarity between pseudogenes and their protein-coding paralogs, it is conceivable that pseudogene-derived ncRNAs may also have a variety of molecular and cellular effects on normal cell growth, human disease, and cancer [12], [19], [35]–[37].

In this study, we have surveyed the landscape of pseudogene transcription across a large number of human tissues and cell lines and begun to explore potential functional and cellular activities of pseudogene ncRNAs from several perspectives. We found that a few thousand human pseudogenes were transcribed and their transcription was overall correlated with increased expression levels and expression diversity of their parental genes. Some pseudogenes, on the other hand, displayed evidence of siRNA production and function, potentially by either interfering with parental gene expression or mediating local epigenetic silencing. Taken together, our results suggest that pseudogene transcription is likely an important process that has provided novel ncRNA elements for modulating the transcriptional fluctuation of protein-coding genes.

## Results

### Identification of transcribed pseudogenes from RNA-Seq data

**Figure 1:**
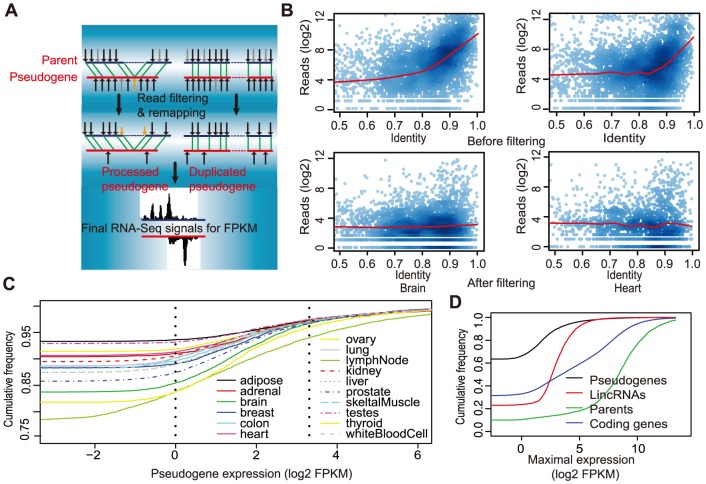
Identification of transcribed pseudogenes from RNA-Seq data. A) A schematic illustration of the key concept of filtering out reads not-uniquely matched to pseudogenes. Black and gray arrows represent perfectly matched and mismatched RNA-Seq reads, respectively, and the matched locations were kept. Yellow arrows represent a read initially put on a processed pseudogene but mapped back to the parent, based on aligning reads to coding sequences, because it is from an exon-exon junction. Green lines denote identical short sequences shared between gene and pseudogene. The left and right cartoons represent processed and duplicated pseudogenes, respectively. The bottom plots final read coverage on a pseudogene (red) and its parent (black), indicating that RNA-Seq signals have largely been resolved. B) Filtering effectively reduces the correlation between the number of mapped reads and sequence identity of a pseudogene to its parental gene. The number of mapped reads (y-axis) within every 200-bp region of a pseudogene is plotted against this region's sequence identity (x-axis) to the parental gene. Representative data for two tissues (brain and heart) were shown (top, before filtering; bottom, after filtering). C) Distributions of transcription values (i.e., FPKMs) of pseudogenes in all 16 tissues (the two vertical dash lines mark 1 and 10 FPKM, respectively). D) Distributions of the maximal FPKMs for lincRNAs, pseudogenes, their parents, and the rest of coding genes.

A major challenge in detecting transcribed pseudogenes is how to map RNA-Seq reads back to their genuine origins when both pseudogenes and their parents are candidates because of their high sequence similarity. The lack of introns may even make a processed pseudogene the preferred candidate for reads originating from exon-exon junctions of the parent. To address these issues directly, we have designed a new computational method to filter out RNA-Seq signals that are likely to have originated from coding genes but can be mapped to pseudogenes due to ambiguity (Fig. 1A, see Material and Methods). For examples, exon-exon junction reads originated from parental genes were removed from pseudogene loci by our method even though their mapping to a processed pseudogene could have a greater alignment score. Without filtering, reads were overwhelmingly mapped to pseudogene regions with >80% sequence identity to their parents, and a positive correlation existed between the number of reads mapped to a pseudogene region and the parental-pseudogene sequence identity (Fig. 1B, top panels). This pattern disappeared after our filtering (Fig. 1B, bottom), indicating that the resulting RNA-Seq signals used for our subsequent pseudogene analyses to be described below were unlikely affected significantly by reads arisen from parental genes. It also suggests that careful filtering of RNA-Seq reads by an extra step of read alignment to the human transcriptome (see Methods) is critical. This has not been explicitly considered in previous identification of transcribed pseudogenes, although in those studies investigators performed other downstream analysis to reduce the contribution of parental transcription signals to pseudogenes [11], [12]. In addition, the majority of current annotated pseudogenes (87.3% out of a total of 11, 205) share <90% sequence identity to their parents (Fig. S1), which would provide on average of ≥5 informative mismatching sites for distinguishing a true pseudogene read from a presumably parent-originating read, given that the length of our RNA-Seq reads is 50–75 bases. In summary, these results indicate that the RNA-Seq signals attributed to pseudogenes by our new computational method are reliable.

To determine pseudogene transcription systematically, we first applied our method to analyze RNA-Seq data from 16 normal human tissues in the Illumina Human Body Map 2 Project, and then to the data from GM12878 and K562 cell lines (see below) in the ENCODE project [16], 38,39. After read filtering, we applied the TopHat/Cufflinks package [40] to compute expression level (in FPKMs, Fragments Per Kilobase of transcript per Million mapped reads). Of the total of 11,205 human pseudogenes annotated by the GENCODE [11], 3,773 (33.7%) and 982 (8.8%) had a value of >1 and >10 FPKM in at least one of the 16 human tissues, respectively (Fig. 1C). By comparison, the corresponding numbers of 77.8% and 47% for protein coding genes are significantly larger (Fig. 1D). To our surprise, the majority of the transcribed pseudogenes were processed pseudogenes (78.6% and 76.2% for FPKM >1 and >10, respectively), even though duplicated pseudogenes would be expected to more likely retain a “functional” promoter. The bias, however, is present in the GENCODE annotation, as 77% of the pseudogenes are annotated as processed, indicating that processed pseudogenes are as likely to produce ncRNAs as duplicated ones. Using the maximal FPKM in the 16 tissues for each pseudogene (or lincRNAs), we found that the median transcription levels of all transcribed pseudogenes and lincRNAs (FPKM >1) were 22- and 11-fold lower than that of protein-coding genes, respectively, indicating that both pseudogenes and lincRNAs were transcribed at significantly lower levels than protein coding genes (Fig. 1D).

### Pattern of pseudogene transcription in normal human tissues

**Figure 2:**
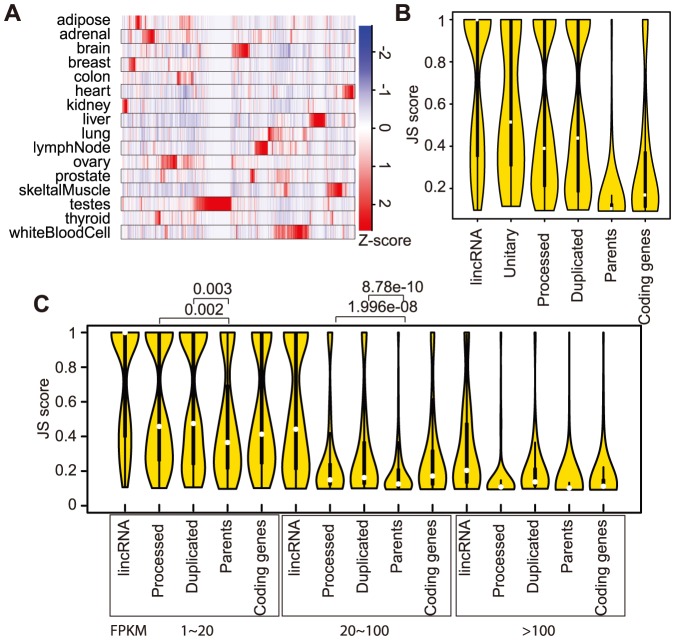
High tissue specificity of pseudogene transcription. A) Heatmap for the transcription levels of 982 highly transcribed pseudogenes (maximal FPKM >10). B) Violin plots showing tissue-specificity JS scores of lincRNAs, transcribed pseudogenes, their parents, and the coding genes without pseudogenes. C) Comparison of JS scores at different transcription levels. The white dots mark median and the thick boxes mark the first and third quartile values.

We next examined pseudogene transcription patterns across normal tissues using two complementary methods. We first compared the expression of pseudogenes that were highly transcribed in at least one tissue (i.e., maximal FPKM >10, n = 982). A hierarchical clustering analysis showed that a subset of pseudogenes was nearly exclusively transcribed in testis (Fig. 2A). White blood cells, ovary, liver, and brain tissues also produced many pseudogene transcripts that were much less abundant in other tissues, but overall every tissue has its own unique set of highly transcribed pseudogenes (see Fig. S2 for examples). The pattern in Fig. 2A was reproducible if pseudogenes of FPKMs either >5 or >1 were clustered (data not shown). In order to better quantify tissue specificity of transcription, we have applied a statistical method recently introduced to characterize lincRNA transcription profiles [41]. The method computes JS (Jensen-Shannon) scores to determine tissue specificity (larger numbers indicating higher tissue specificity; see Methods). We determined JS scores for the pseudogenes of maximal FPKM>1, which we considered as “transcribed pseudogenes” (Table S1, n = 3,773). The results indicated that pseudogene transcription (from all three types: processed, duplicated and unitary) exhibited significantly higher tissue specificity than the expression of protein coding genes, measured against either all parental genes or all protein coding genes without a pseudogene relative (referred to hereafter as “coding genes”) (*p*<2.2e-16, Wilcoxon test) (Fig. 2B). LincRNAs showed the highest tissue specificity by this measurement (Fig. 2B). The tissue-specific JS scores were negatively correlated to expression values (Fig. 2C).

To determine to what extent the high JS scores for pseudogenes could be explained by their low transcription, we computed JS scores for randomly selected coding genes with maximal FPKMs matched to those of pseudogenes. We found that the JS scores of processed pseudogenes remained lower than their expression-matching coding genes (p<0.005, Wilcoxon test), but for duplicated pseudogenes the difference was more significant at high expression level (Fig. S2), suggesting that we cannot fully untangle the underlying correlation. Low JS scores, however, were unlikely a result of a few mapped reads in few tissues, since JS scores from full RNA-Seq datasets were highly similar and correlated to those computed with only one half of the RNA-Seq data (Fig. S2). Interestingly, parents of all transcribed pseudogenes also displayed lower tissue-specific transcription than coding genes without any pseudogene relatives (Fig. 2B,C), which is probably explained by the fact that housekeeping genes are a major source of processed pseudogenes [4]. In light of this and to reduce potential systematic bias of pseudogenes from broadly expressed parents, we have selected only transcribed pseudogenes (n = 1,270) derived from *parents* that had a JS score >0.1 for all studies described below unless mentioned otherwise, which effectively excluded nearly all (n = 745) pseudogenes derived from ribosomal protein genes.

In summary, the above results for the extent of pseudogene transcription and their tissue expression pattern are consistent with previous reports [8], [9], [11], [42]–[44], suggesting that our analyses and the results that will be described below reflect general properties of pseudogene ncRNAs but not specific to our set of transcribed pseudogenes. We should also mention the primary goal of the current study is not simply to compile a list of all human transcribed pseudogenes, but to characterize those that have robust and consistent evidence of transcription.

### Tissue specificity *vs* transcription factor binding

To explore transcriptional regulation potentially contributing to tissue specificity, we examined the number of transcription factor (TF) binding events in the promoters (−2 kb to transcription start sites (TSS)) of pseudogenes and protein coding genes, using the integrated ChIP-Seq data from the ENCODE project [16]. More specifically, the data contain 2,750,490 ChIP-seq peaks merged from 690 ChIP-seq datasets representing the genomic occupancy of 161 unique regulatory factors (both generic and sequence-specific factors) in 91 human cell types. We found that both pseudogenes and genes of lower tissue specificity (JS<0.2) were bound by more transcription factors (multiple binding events of the same factor were counted as one) than their counterparts with higher JS scores (JS≥0.2) (Wilcoxon test, *p*<0.02 and <2.2e-16 for pseudogenes and genes, respectively; data not shown). The difference remained statistically significant (p values <0.04) when the JS score cutoff was set to 0.25 or 0.35. In addition, changing the promoter definition slightly from ±2 kb to ±5 kb or ±10 kb produced similar statistics (e.g., p<0.01 and <5e-14 for pseudogenes and genes when ±5 kb was used). Since the data capture a mixture of TF events in 91 cell types, this result suggests that pseudogenes transcribed more broadly contain more potential regulatory sites, but the functional importance of this observation needs further investigation.

### Positive transcriptional relationship between pseudogenes and coding genes

**Figure 3:**
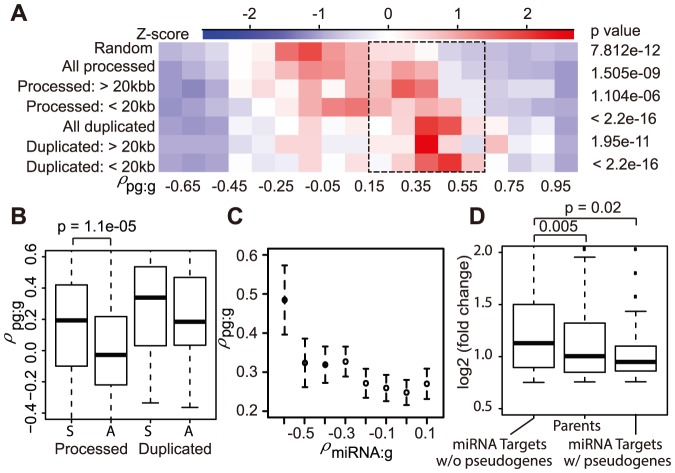
Transcriptional correlations (ρ_pg:g_) between pseudogenes and their parents. A) A heatmap for distribution of ρ_pg:g_, including data from separation of processed and duplicated pseudogenes into two groups based on the presence of a coding gene within 20 kb. The coefficients between transcribed pseudogenes and randomly chosen coding genes (top) were used as a control for p-value estimation. Colors represent relative numbers of pseudogenes in each ρ_pg:g_ range (in Z-score transformation). B) Pseudogenes transcribed in the sense direction (S) exhibited higher ρ_pg:g_ than those in the antisense (A). C) The transcriptional correlation between pseudogenes and their parents (ρ_pg:g_) is inversely correlated to the transcriptional correlation between miRNAs and their putative targets (ρ_miRNA:g_). Genes were binned on their ρ_miRNA:g_ values (x-axis) and then the mean and standard deviation of ρ_pg:g_ (y-axis) for each group of genes was plotted. D) Expression of parental genes targeted by miRNAs was less affected by miRNA KD than the targeting genes without pseudogenes. Only genes in response to KD (up >1.3 fold) were analyzed here. Y-axis shows the fold change of KD over control. The miRNA targets were experimentally determined by the CLASH analysis [49]. The middle line in the boxplots mark median and the box lines mark the first and third quartile values (same for boxplots below).

The evidence of pervasive pseudogene transcription is compelling, but more important questions are what kinds of biological functions pseudogene ncRNAs can have. Note that the term “biological function” in this report is used in a loose sense, whereas “biochemical activity” may arguably be more appropriate, in accordance with the source of our experimental data and the computational nature of our work. The first obvious question is how pseudogene and parent gene transcription are related, as this information may shed light on how pseudogenes could regulate their most conceivable targets. To this end, we computed the Spearman rank correlation of the 16 tissue transcription levels for each of the 1,270 pseudogene-parent pairs (ρ_pg:g_). The resulting correlation coefficients for both processed and duplicated pseudogenes showed a distribution that was deviated from the theoretical normal distributions (*p* = 0.05, Kolmogorov-Smirnov (KS) test) and biased towards positive numbers (ρ_pg:g_ median  = 0.42 and 0.12 for duplicated and processed pseudogenes, respectively, Fig. 3A). The skew was statistically significant, when compared to the distribution of the ρ between transcribed pseudogenes and randomly selected coding genes (Fig. 3A and Fig. S3). In addition, 128 and 95 of the positive ρ_pg:g_ values for processed and duplicated pseudogenes were statistically significant (p<0.05). Since some pseudogenes are close to their parents on chromosomes (e.g., those from tandem duplications) and adjacent genes tend to be co-regulated [41], we computed and used the chromosomal distances of transcribed pseudogenes to the nearest coding gene to separate transcribed pseudogenes within 20 kb of a gene (“group t1”; n = 712 and 236 for processed and duplicated, respectively) from the rest (“group t2”; n = 167 and 78 for processed and duplicated, respectively). We found that ρ_pg:g_ values for the t2 group remained skewed to positive for both processed and duplicated pseudogenes (ρ_pg:g_ median = 0.42 and 0.41 for group t1 and t2 duplicated, and 0.08 and 0.25 for processed pseudogenes; Fig 3A). Interestingly, this breakdown indeed revealed that group t2 processed pseudogenes showed even larger correlations with their parents (Wilcoxon test, p<0.002). These results suggest that our observation of positive ρ_pg:g_ values for most pseudogenes did not arise from co-regulation of pseudogenes and their parents due to their close chromosomal proximity. We noted that the difference between t1 and t2 processed pseudogenes remained significant when longer distances were applied (p<0.002, 0.008 and 0.02 for 20 kb, 50 kb and 100 kb, respectively). In summary, our results indicate that pseudogene transcription is positively correlated with the expression of their parents.

Although our observation is based on correlation, it is consistent with the ceRNA hypothesis [45] that pseudogene ncRNAs can act as miRNA sponges and thus positively regulate the expression of their parents by titrating cellular miRNAs that are otherwise targeted to protein coding genes. This novel ncRNA functional mechanism was demonstrated elegantly in the study detailing the miRNA decoy functions of two pseudogenes, *PTENP1* and *KRAS1P* [28]. To explore the generalization of this mechanism globally, we examined how the number of miRNA sites within a pseudogene influenced its transcriptional correlation with its parent. Although pseudogenes with more putative miRNA binding sites exhibited on average larger ρ_pg:g_ values than those with fewer sites, the difference is not significant if the pseudogene lengths were factored in (data not shown).

Three additional lines of evidence, however, supported the idea that the positive correlation between pseudogene ncRNAs and parental mRNAs could be due to miRNA binding competition, at least partially. First of all, the competing interactions are expected to be stronger if pseudogene ncRNAs are sense to the parental mRNAs. Since the transcriptional direction of a pseudogene could be different from the annotated one and our current RNA-Seq data did not contain strand information, we used strand-specific RNA-Seq dataset (GEO: GSE32307) from a previous study [46] to infer transcription direction (see Methods), with the assumption that the strand of pseudogene transcription maintains the same from one tissue/cell to another. When the resultant information was included, interestingly but as predicted, processed pseudogenes generating sense ncRNAs had a significantly higher ρ_pg:g_ values than those producing antisense ncRNAs (median of 0.19 and −0.03 for sense and antisense, respectively), while duplicated pseudogenes followed the same trends (median of 0.34 and 0.19 for sense and antisense, respectively; Fig. 3B). Secondarily, we analyzed the transcriptional profiles of parent genes, pseudogenes and miRNAs by integrating a miRNA microarray expression dataset collected for 15 out of 16 analyzed tissues (no data for white blood cells) in a previous study [47]. For every parental gene, we computed its expression correlation (ρ_miRNA:g_) to each of the miRNAs that it can putatively bind. Likewise, we calculated ρ_miRNA:pg._ This produced three-way pairwise correlations. To plot the data, we binned genes to groups based on ρ_miRNA:g_ numbers (Fig. 3C, x-axis) and then for each group we computed the mean (and standard deviation, y-axis) of ρ_pg:g._ This analysis revealed a negative correlation between ρ_pg:g_ (i.e., the co-transcriptional relationship of pseudogenes and their parents) and ρ_miRNA:g_ (i.e., the co-transcriptional relationship of miRNAs and parents) (Fig. 3C, *p* = 1.5e-06, r = −0.1). This pattern implies that the miRNA sponge effect of a pseudogene ncRNA is potentially more significant, manifested as a large and positive ρ_pg:g_, if the shared miRNA shows a larger inhibition to the parent, indicated by a small and negative ρ_miRNA:g_, further supporting the idea that a miRNA sponge effect could partially contribute to the observed transcriptional correlation between pseudogenes and their parents, especially in the cases when miRNA regulatory effects were large (i.e., ρ_miRNA:g_<−0.5, Fig. 3C).

To seek additional experimental support, we examined a total of 19,184 high confident *in vivo* miRNA target sites in HEK293 cell line as determined by AGO and TNRC6 occupancy using the PAR-CLIP technology [48]; AGO and TNRC6 are two key components of miRNA-containing ribonucleoprotein complexes. Out of the 1,270 pseudogenes, 18 were found to contain at least one AGO/TNRC6 binding site, 11 of which located towards the end of pseudogenes, with an additional 10 pseudogenes containing sites in their 1 kb flanking regions (binomial test, p<0.05). Even more interestingly, our reanalysis of the microarray expression data upon knockdown (KD) of the top 25 different miRNAs expressed in HEK293 [48] revealed that, among the genes up-regulated >1.3-fold by KDs, the parents of pseudogenes and especially those targeted by the 25 miRNAs showed smaller increases than the coding genes that were targeted by these miRNAs but did not have a pseudogene relative (Fig. 3D). The different responses to miRNA KDs remained if 1.2-, 1.5-, or 2-fold change was applied (data not shown). For this analysis, the miRNA targets were extracted from the experimentally determined miRNA-mRNA interactions by the CLASH analysis [49]. Notably, the CLASH study reported that 4.9% of the identified miRNA-RNA interactions were mapped to pseudogenes [49]. These results provide strong evidence for a miRNA sponge effect of pseudogene ncRNAs. We provide the five pseudogenes that were most likely to function as miRNA decoys in Table 1, including *PTENP1,* and other candidates in Table S1.

**Table 1:** Top pseudogene candidates of three different types of predicted functional potentials (ND, not determined). The full lists can be found in Table S1.

Pseudogene	Genomic Location	Parental gene	Transcribed strand	ρ_pg:g_	Note
**A. miRNA decoy**					
*PTENP1*	chr9:33673502-33677497 (−)	*PTEN*	sense	0.87	Compete with *PTEN* for miRNA binding [28].
*FAM92A1P1*	chr15:41455322-41456695 (+)	*FAM92A1*	sense	0.80	
*MYLKP1*	chr3:75377700-75388222 (−)	*MYLK*	sense	0.94	Promote cell proliferation [100], but miRNA involvement unknown.
*CROCCP3*	chr1:16802411-16817802 (−)	*CROCC*	sense	0.70	
*ABCC6P2*	chr16:14914649-14918526 (−)	*ABCC6*	NA	0.50	
*RP11-321E8*	chr7:63929563-63931031 (+)	*ZNF680*	sense	0.39	
**B. siRNA repressing coding gene**					
*ψPPM1K*	chr4:89179936-89180414 (−)	*PPM1K*	antisense	ND	Target both *NEK8* and *PPM1K* gene and suppress cell growth [30]. Not annotated by GENCODE.
*ATP8A2P1*	chr10:37537046-37604729 (+)	*ATP8A2*	antisense	−0.22	KD inhibited cell proliferation [12]
*HMGA1P7*	chr6:134435733-134436628 (−)	*HMGA1*	antisense	−0.25	
*CNN2P1*	chr22:30442265-30443182 (−)	*CNN2*	antisense	−0.32	
*RP11-553K8.3*	chr1:198648263-198649162 (−)	*PEBP1*	NA	−0.56	
*MSNP1*	chr5:25909612-25911343 (+)	*MSN*	sense	−0.5	Antisense ncRNA was reported [19]
*RP11-159C21.4*	chr1:53237865 -53238320(−)	*RPS13*	sense	0.52	
**C. siRNA mediating H3K9me3 enrichment**					
*MTND4P12*	chr5:134262350-134263726(−)	*MTND4*	both	ND	
*RP5-857K21.6*	chr1:566454-567996(+)	*MT-CO1*	sense	ND	
*SDHAP2*	chr3:195384967-195412775(+)	*SDHA*	ND	0.54	
*FTLP3*	chr20:4004564-4005091(+)	*FTL*	sense	ND	
*RP11-7G23.4*	chr9:45729709-45730417(+)	*FAM27A*	ND	ND	

### Pseudogene transcription increases the mRNA abundance of their parental genes

**Figure 4:**
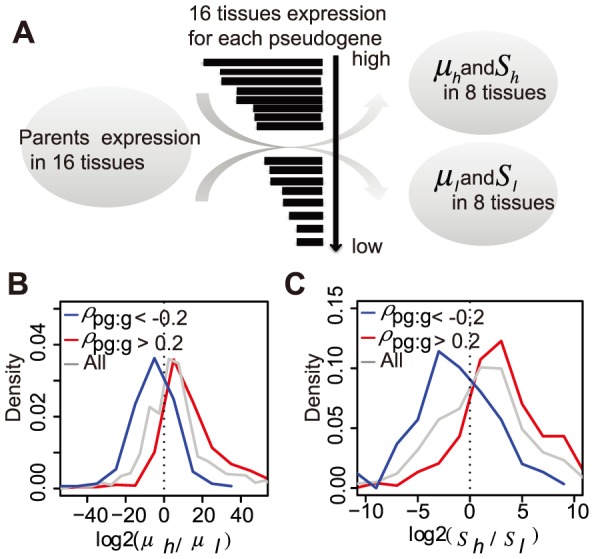
Pseudogene transcription increases the mean and variance of parental gene expression. A) A cartoon illustrating the computational procedure. For each pseudogene, we computed the means (μ_h_ and μ_l_) and variances (S_h_ and S_l_) of its parental gene expression values in the 8 tissue samples with more pseudogene transcripts and the 8 with fewer pseudogene transcripts. Distribution of mean (B) and variance (C) differences for all transcribed pseudogenes, pseudogenes with positive (ρ_pg:g_>0.2) and negative (ρ_pg:g_<−0.2) transcriptional correlation with their parents.

Having demonstrated the positive transcriptional relationship between pseudogenes and their parents, we next set out to confirm that parental genes were indeed expressed at higher levels in the same tissues where more pseudogene ncRNAs were found. First of all, using FPKM >1 as a simple threshold for calling the presence of pseudogenes in a tissue, we found that for >70% of cases the parent was also detected with >1 FPKM in the same tissue in which a pseudogene was transcribed. Next, we split the 16 tissues into two groups of eight each by the transcriptional levels of a pseudogene, and then examined how its parent gene was differently expressed between the two groups (Fig. 4A). Note that this splitting was performed for each of the 1,270 pseudogenes independently. The resulting between-group difference in both means and variances for all pseudogenes are shown in Figure 4. Q-Q plot analysis indicated that both the mean differences and variance differences exhibited a non-normal distribution (Fig. S4). More interestingly, for the parent-pseudogene pairs with positive ρ_pg:g_, both the means and variances of parental gene expression were greater in the tissues where pseudogene ncRNA levels were higher (Fig. 4B,C, red lines). For those pairs of negative ρ_pg:g_, the trends were reversed (Fig. 4B,C, blue lines). These results suggest that pseudogene transcription may play a role in both the level and diversity of their parental gene expression, but not to a great extent. This hypothesis was further supported by the comparison of gene expression across all 16 tissues (without splitting) for parents whose corresponding pseudogenes were transcribed at different levels (Fig. S4).

### Pseudogene derived small RNAs and their potential roles

**Figure 5:**
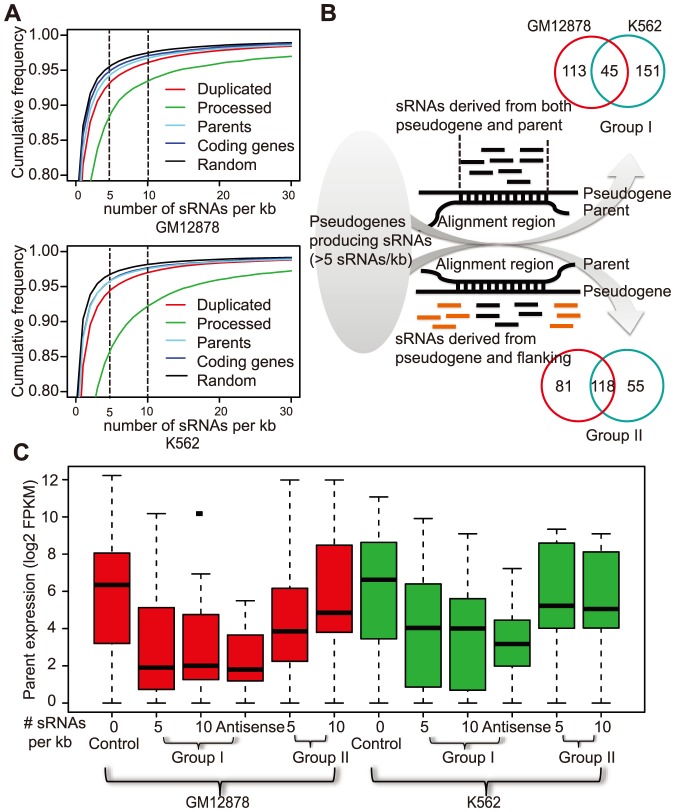
Pseudogene-derived sRNAs and their relationship to parental gene repression. A) Processed pseudogenes had higher sRNA read densities than any other annotated genomic elements and randomly chosen genomic regions in both GM12878 and K562 cell lines. B) Pseudogenes with mapped sRNA reads (≥5 reads per kb) were separated into two groups based on the abundance of sRNA reads in the adjacent non-pseudogene regions (±1 kb, orange). Group I was considered to produce sRNA interactively with their parents while group II produced sRNA independently. Venn diagrams show the data comparison between GM12878 (red) and K562 (green). C) The parental genes of group I pseudogenes showed significantly lower expression than either those of the pseudogenes without sRNA (control) or those of the group II pseudogenes, in both GM12878 (red) and K562 (green). The parents of antisense transcribed pseudogenes (>5 sRNA/kb) exhibited even lower expression. The same trends held when the analysis was carried out for pseudogenes with >10 sRNA/kb. Parents not expressed in the 16 normal tissues (i.e., FPKM = 0) were not included in these plots.

The above studies address the potential roles of pseudogenes as a novel source of long ncRNAs (lncRNAs), but pseudogene transcripts can also be used to produce small RNAs, which can potentially execute a variety of functions [50]–[53]. In particular, it has previously been suggested that pseudogene ncRNAs may form double stranded RNAs (dsRNAs) with cellular mRNAs from their parental genes, and the dsRNAs can in turn be processed by the cellular siRNA generation machinery to produce functional small interference RNAs (siRNAs) [2], [15], [54]. This has been shown experimentally in mouse oocytes [22], [23], *Trypanosoma brucei* [18], and recently in human hepatocellular carcinoma [30]. To explore this, we analyzed the sequencing data of small RNAs (<200 bp) from two cell lines, GM12878 and K562, from the ENCODE project [39], [55], and relate sRNA production with gene expression in these two cell lines, since small RNA-Seq data have not become available for the 16 normal human tissues used by the Body Map project. We first compared the overall densities of small RNAs mapped to pseudogenes and coding genes. The data indicated that processed pseudogenes exhibited significantly higher sRNA density than duplicated pseudogenes and coding genes in both GM12878 and K562 (Fig. 5A; Wilcoxon test, p<2.2e-16;). Notably, parents of pseudogenes appeared to have greater sRNA production capacity than the other coding genes.

To investigate the potential functions of pseudogene-derived sRNAs, we analyzed pseudogenes that produced relatively large numbers of sRNAs (average >5 sRNAs per kb in the exonic regions of pseudogenes; information and data about their transcription in the aforementioned 16 normal human tissues were not considered here). Two subsets of such pseudogenes were selected for comparison, based on whether sRNAs were also detected in the flanking regions (Fig. 5B), with the assumption that they represent two fundamentally distinct biogenesis/functional mechanisms (see Method for details). We reasoned that “group I” likely represents pseudogenes that produced sRNAs *via* dsRNA intermediates formed either between pseudogene ncRNAs and parental mRNAs, or in the hairpin loops of pseudogene ncRNAs, whereas “group II” pseudogenes probably can generate sRNAs independent of their parents in a manner similar to repeats and transposons located at heterochromatin regions (Fig. 5B). Interestingly, comparison of data in GM12878 and K562 indicated that sRNAs from the group I pseudogenes were more likely to be cell specific than sRNAs from group II (Fisher exact test, *p*<2.2e-16). We hypothesized that the parental genes of group I pseudogenes would be expressed at lower levels than the parents of both group II pseudogenes and the pseudogenes that did not produce sRNAs (“Control” in Fig. 5), based on previous reports that pseudogene-derived siRNAs could function as endo-siRNAs and reduce parental gene expression in mouse oocytes [22], [23] and the assumption that the biogenesis of this group of sRNAs from the dsRNAs formed between parental mRNAs and pseudogene ncRNAs would result in sRNA detection in both genes and pseudogenes. As shown in Fig. 5C, the data are indeed consistent with this hypothesis; in both GM12878 and K562, the parents of group I pseudogenes exhibited the lowest expression (Wilcoxon test, *p*<0.05 for all comparisons). This trend persisted if the threshold was changed to >10 sRNA per kb (Fig. 5C), although smaller numbers of pseudogenes would meet this criterion. While siRNA biogenesis and functional mechanisms are complex, and the exact molecular process in human cells remains unclear, we did observe that 8% and 13% of group I pseudogenes exhibited evidence of either antisense or both stranded transcription, respectively. The strand information was inferred as described above, because neither the small RNA-seq nor the RNA-seq data from GM12878 and K562 recorded transcription direction. As expected, the parents of the antisense transcribed group I pseudogenes showed further decreased expression (Fig. 5C). In Table 1, we list the five pseudogenes that were most likely to produce functional antisense siRNAs, including*ψPPM1K* [30]. We should, however, caution that the precursors for sRNAs could be transcribed distinctly from the lncRNAs detected in the strand-specific RNA-Seq datasets. In addition, some of these sRNAs could be derived from the hairpin RNA loops in the pseudogene ncRNAs, as reported previously [22]–[24], [30], but more studies are required to address this in the future.

For the small RNAs generated from pseudogenes independently of their parents (i.e., inferred from sRNA presence beyond the parent-pseudogene aligned regions; group II in Fig. 5B), we are interested in their potential involvement in recruiting chromatin modifiers and mediating epigenetic silencing. This is motivated by the requirement of piRNAs for repressing transposons [56], the involvement of endo-siRNAs in repressing long interspersed nuclear element-1 (LINE-1) activity [57], [58], and the facts that (a) many sRNAs in our dataset were mapped to repetitive elements in the human genome (data not shown), (b) siRNAs from both pseudogenes and transposons in plants have been implicated in RNA-directed DNA methylation, and (c) mammalian ncRNAs have emerged as key epigenetic regulators [59], [60]. Very interestingly, we found that sRNAs from the group II pseudogenes (median size 24–27 bp) were 2–6 bp longer than the group I sRNAs; this difference was significant in both GM12878 and K562 but a greater difference was seen from K562 data (KS test, p<0.0002; Fig. S5), providing an empirical support to our discrimination of the two pseudogene groups. Note that it has been shown that pseudogenes and repeats derived sRNAs involved in epigenetic silencing in plants were ∼24 bp [24].

**Figure 6:**
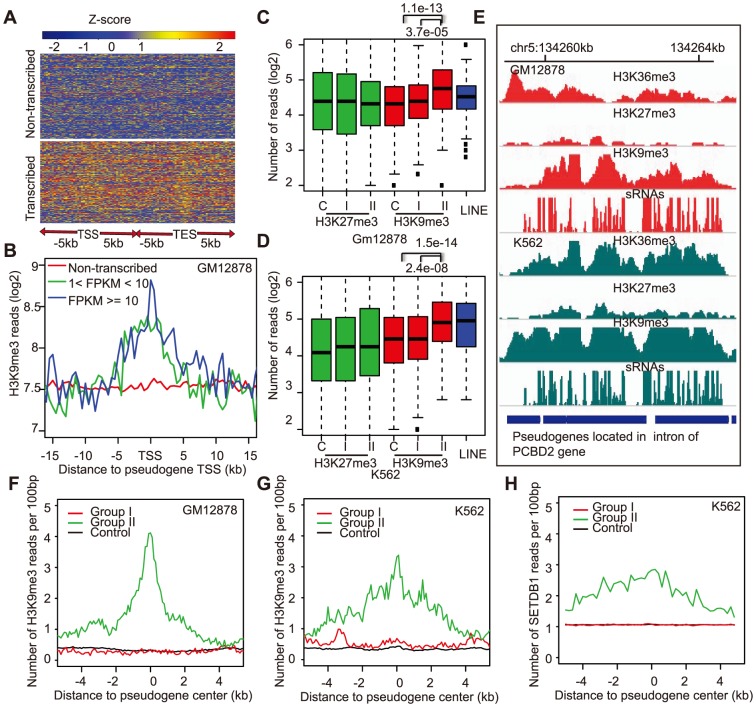
Enrichment of H3K9me3 modification at transcribed pseudogene loci. A) Heatmap of H3K36me3 near the transcription start sites (TSS) and transcription end sites (TES) of transcribed (bottom) and non-transcribed pseudogenes (top). The color scheme is based on column-based normalization data in GM12878, whereas each row is a pseudogene. B) Transcription level dependent enrichment of H3K9me3 at transcribed pseudogenes. Y-axis shows the average number of H3K9me3 ChIP-Seq reads per 500 bp. C) & D) The level of H3K9me3 (red) but not H3K27me3 (green) was significantly higher at group II pseudogenes (Fig. 5) than at group I pseudogenes or at pseudogenes loci producing no sRNAs (“C”, controls). The H3K9me3 level at a randomly selected set of LINE (blue) was also plotted as positive controls. Y-axis plots ChIP-Seq reads at pseudogene bodies, normalized to per 500-bp sequences. E) The densities of H3K36me3, H3K27me3, and H3K9me3 ChIP-Seq reads and sRNA-Seq reads at a region with multiple pseudogenes derived from a gene encoding NADH dehydrogenase. F–H) The average ChIP-Seq profiles, anchored on pseudogene centers, of H3K9me3 in GM12878 (F) and in K562 (G) and of SETDB1 in K562 (H) for the three groups of pseudogenes. Y-axes show the average numbers of ChIP-Seq reads per 100 bp.

We began with a comparison of transcribed *vs* non-transcribed pseudogenes with respect to several types of histone modifications, using the ChIP-Seq and RNA-Seq data only from GM12878 and K562. Globally, we observed a clear distinction between transcribed and non-transcribed pseudogenes with respect to H3K36me3 (Fig. 6A), a histone modification associated with transcription elongation. This pattern provided strong epigenetic support for our method to reliably identify transcribed pseudogenes, as the H3K36me3 enrichment extended to pseudogene adjacent regions. Other active chromatin marks, including H3K4me3, H3K4me1, H3K4me2, H3K9ac and H3K27ac, were also significantly more enriched in transcribed pseudogenes than non-transcribed ones (Fig. S6), while the repressive marker H3K27me3 was depleted, in agreement with results from the GENCODE study [11]. To our surprise, H3K9me3, a repressive chromatin mark most often found in transcriptionally inactive repeats or heterochromatin [61], [62], was more abundant in transcribed pseudogenes than the non-transcribed ones (Fig. S6, *p* = 3.7e-05 and 2.4e-08 for GM12878 and K562, respectively). Furthermore, the extent of H3K9me3 within 15 kb of the transcription start sites showed a clear enrichment that was dependent on pseudogene transcription levels (Fig. 6B). The association of transcribed pseudogenes with H3K9me3 enrichment was not restricted to cancer cell lines, since 9.3%, 7.3% and 4% of the transcribed pseudogenes (FPKM >1) in adipose, liver and skeletal muscle, respectively, intersected with the H3K9me3 enriched regions determined in a recent study of chromatin states for multiple human tissues [63].

In order to address whether H3K9me3 enrichment was related to pseudogene-derived sRNAs, we compared the H3K9me3 levels between the two groups of sRNA-producing pseudogenes (Fig. 5B). As shown in Figure 6, the group II pseudogenes, which likely produced sRNAs independently from their parents, exhibited a significantly higher level of H3K9me3 than group I pseudogenes, as well as those pseudogenes without detectable sRNAs (“Control”), in both GM12878 (Fig. 6C,F) and K562 cells (Fig. 6D,G). This distinction was not seen for H3K27me3 (Fig. 6C,D; green), indicating that our observation was specific to H3K9me3 and not due to either overall transcription repression or ChIP-Seq experimental artifacts. An example is illustrated in Fig. 6E, which shows broad H3K9me3 enrichment around a region on chromosome 5 containing multiple pseudogenes that produced an extensive number of sRNAs. To further support the idea that the pseudogene sRNA-related H3K9me3 enrichment was independent of the dsRNAs formed with parental mRNAs, we analyzed unitary pseudogenes, which do not have obvious paralogous coding genes. We found that unitary pseudogenes with detectable sRNAs (n = 28) also had increased H3K9me3 levels when compared to those (n = 381) with no sRNAs (*p* = 6.7e-7 for GM12878 and *p* = 0.0052 for K562). We hypothesize that these results suggest pseudogene-derived sRNAs can play an active role in the establishment of broad but local silencing chromatin environment for repressing pseudogene transcription, a phenomenon that has been documented in yeast and plants [64] (see Discussion). If so, one would expect similar enrichment of H3K9 methyltransferase (e.g., SETDB1) in the group II pseudogenes. Using the only SETDB1 ChIP-Seq data currently available (for K562), we found that the SETDB1 ChIP-Seq signal was indeed significantly higher at the group II pseudogenes (Fig. 6F). To add further support to the potential existence of sRNA-mediated chromatin repression in human cells, we found that LINEs with more (>5/kb) sRNAs were marked by significantly higher levels of H3K9me3 than LINEs with hardly any sRNAs (<1/kb) in both GM12878 and K562 cells (Wilcoxon test, p<2.2e-16). Higher level of H3K9me3 at the group II pseudogenes, however, is not a result of more repetitive elements within them. Neither were repeats (e.g, LINEs, LTRs, and ALUs) enriched at transcribed pseudogenes (in comparison to adjacent genomic regions; Fig. S6C), nor was there a higher density of repeats in the group II than the group I pseudogenes, which were analyzed on either full pseudogene bodies or with 5-, 10- or 25-kb extensions to the flanking regions (all p values >0.2, Wilcoxon test).

### Evolutionary constraints on transcribed pseudogenes

**Figure 7:**
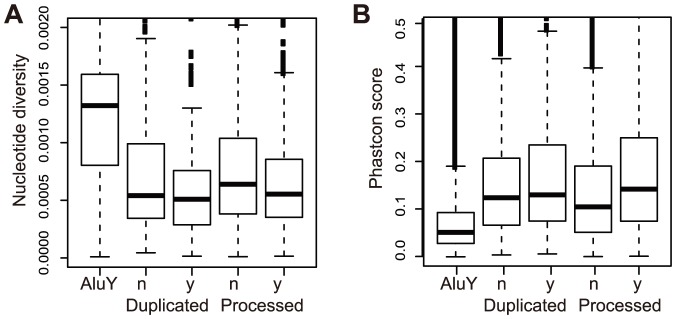
Selection constraints on transcribed pseudogenes. Comparison of nucleotide diversities in human population (A) and cross-species conservations (B) between non-transcribed (‘n’) and transcribed pseudogenes (‘y’). AluY, a young repeats that emerged recently in primates, was used as control. For duplicated pseudogenes, the median diversities for transcribed and non-transcribed are 0. 00051 and 0.00054 (p<0.02, Wilcoxon test), the values for processed pseudogenes are 0.00055 and 0.00064 (p<3e-06, Wilcoxon test).

Our findings suggest that many human pseudogenes are transcribed and the transcripts exhibit evidence for various biological activities. It is possible that the human transcriptome and its regulation are sufficiently robust to tolerate the small perturbation introduced by pseudogene transcription. If so, transcribed pseudogenes would not show significantly different evolutionary constraints compared with non-transcribed ones. Therefore, we analyzed nucleotide diversity (based on two population datasets from the HapMap project) and cross-species conservation (based on 46 way phastcon scores) within pseudogenes. The data show that transcribed pseudogenes exhibit significantly higher evolutionary constraints than non-transcribed ones, as suggested by the lower degree of polymorphism and greater phastcon scores (Wilcoxon test, p<0.001, Fig. 7). While the assessment of sequence conservation in pseudogenes could be confounded by difficulties in cross-genome alignment and ortholog assignment, the nucleotide diversity data derived from two distinct human populations were highly similar and correlated (Fig. S7), indicating that our result is not a simple consequence of some genomic sequences that have only recently lost their protein coding functions. In addition, extremely young pseudogenes, such as the human specific ones [65], [66], would not be called as transcribed pseudogenes by our method. Therefore, we conclude that some transcribed pseudogenes experienced evolutionary constraints and likely have cellular functions, consistent with the results described above. This conclusion is consistent with similar finding by the GENCODE group [11] and is in line with a recent study reporting that some unitary pseudogenes may have lost their coding potential but retain their ncRNA function [67].

## Discussion

The prevalence of pseudogenes is a key feature of the human genome and other mammalian genomes, but the potential functional importance remains unclear. In this study, we have found several thousand pseudogenes transcribed at different levels across human tissues and cell lines based on RNA-Seq data. Our detailed characterizations of transcribed pseudogenes demonstrate that pseudogene ncRNAs share many features with lincRNAs, including high tissue specificity and low abundance. Our study of the transcriptional relationship between pseudogenes and protein-coding genes suggests that pseudogene transcripts could play important roles in regulating gene expression directly by at least two distinct mechanisms: small RNA interference or miRNA competition.

One confounding factor in detecting pseudogene transcription is the high sequence similarity between pseudogenes and their coding paralogs. We believe that our approach has reduced the possibility of mistakenly assigning RNA-Seq reads originated from coding genes to pseudogenes, but some level of ambiguity due to sequencing errors or polymorphism, etc., probably remains. The fraction (∼1/3) of human pseudogenes found to be transcriptionally active in the current study is consistent with previous estimates [8]–[12], which supports the reliability of our method. Furthermore, the enrichment of active histone modifications near our transcribed pseudogenes is good evidence for *bone fide* pseudogene transcription, since the regions analyzed for histone modifications include sequences immediately adjacent to pseudogenes and thus not shared between pseudogenes and their parents (Fig. S6). Nevertheless, more computational and experimental approaches are certainly required to fully address this issue, for example, by the analysis of longer RNA-Seq reads or full-length sequencing data from single RNA molecules [68], or the usage of a probabilistic method for resolving ambiguously mapped reads. Although our approach contains a read-filtering step that was not used in previous methods [11], [12], a direct comparison of these methods for their performance in identifying transcribed pseudogenes is beyond the scope of current study. Nevertheless, we have identified 493 (56%) of the 876 transcribed pseudogenes annotated by the GENCODE team [11]; moreover, of the subset (344) based on the same Body Map data, we found 266 (77%). Those missed by our method typically had a very small FPKM values. Likewise, we identified 822 (62%) of the 1,326 pseudogenes that were actively transcribed in various cancers [12] and present in the GENCODE pseudogene annotation. In terms of validation, 321 of our transcribed pseudogenes were included in the RT-PCR-Seq experiments conducted by the ENCODE project and transcription for 268 (83%) was confirmed [11].

We should point out that pseudogenes with sequences identical to their parents would not have any mapped RNA-Seq reads and thus would be treated as non-transcribed by our current approach, even though they may well be transcribed in the tissue samples. Another caveat of our approach is potential underestimation of expression levels for certain pseudogenes, because some *bona fide* pseudogene-originating reads could be removed. Although further detailed assessment is needed when new computational algorithms or experimental technologies for better quantifying pseudogene expression become available, we believe these factors have not introduced significant bias to our results. For instance, we examined the expression levels of 161 pairs of pseudogenes, in which each pair were derived from the same parental gene but they had distinct identities to the parent. These pseudogene pairs exhibited no expression difference in all the 16 tissues except two (lung and lymph node; paired t-test, multiple test corrected p<0.05), with larger FPKMs for the pseudogenes that were more similar to their parents. We also found that pseudogenes with higher sequence similarity to their parents had lower JS scores and greater ρ_pg:g_ values, indicating that young pseudogenes are more widely transcribed than the old ones, perhaps due to less decay of their promoters.

Our study has systematically explored the potential functional activities of transcribed pseudogenes from several perspectives. Our finding of transcription-dependent H3K9me3 enrichment in some pseudogenes suggests that pseudogene-derived sRNAs may play a role in modulating epigenetic repression of pseudogene transcription, probably by the same molecular mechanism(s) underlying sRNA-mediated heterochromatin formation [69], [70]. While this kind of function has been more extensively studied for plant pseudogenes and found to involve both RNA-dependent RNA polymerases (RdRP) and the RNA-directed DNA methylation (RdDM) pathway [64], [71], it has not been determined whether a similar RdRP-dependent process is also required for repressing mammalian pseudogenes or retrotransposons. Nevertheless, dsRNAs can potentially be generated from human pseudogene ncRNAs since a mammalian enzyme with RdRP activity was identified recently [72], [73], and small RNAs derived from pseudogene ncRNAs with inverted complementary sequences have been reported [22], [23]. In addition, our reanalysis of small RNA expression data before and after Dicer KD (GEO: GSE31069) [74] found a reduction of sRNAs for ∼80% of the 360 pseudogenes that contained at least one uniquely mapped sRNA read in the control treatment of a MCF-7 cell line, including the one shown in Fig. 6E. For the pseudogenes with >5 sRNA reads, all had fewer sRNA reads in Dicer KD. This observation suggests that the biogenesis of pseudogene-derived sRNAs may be affected by Dicer in human cells. Note that mammalian Dicer has previously been implicated in the formation of centromeric heterochromatin [75], [76]. The pseudogene-derived sRNAs can then potentially suppress pseudogene transcription by various means [22], [51], [77], [78], such as those described previously for epigenetic repression in other contexts: promoter-associated RNAs directing epigenetic silencing complexes to their targets [79], L1-derived siRNAs suppressing L1 retrotransposition [57], *Xist* modulating X chromosome inactivation [80], or piRNA targeting transposon repression [56]. Although our data cannot distinguish between these possibilities, our findings suggest that a feedback loop could be involved in transcriptional silencing of pseudogenes. Perhaps this is an active repression mechanism that a host genome uses to suppress pseudogene transcription; consequently, this leads to our observation that pseudogene transcription overall occurs at a very low level, whereas low transcription is needed for the recruitment of epigenetic modifying complexes. Indeed, in both yeast and plants, the sRNAs and chromatin structure constitute a feed-forward loop: sRNAs are needed for establishing specific chromatin modifications, while the distinct chromatin structure is required for the recruitment of cellular machinery for sRNA generation [81]. Moreover, we observed that the pseudogene-derived sRNA mediating repression could also repress neighboring genes in addition to pseudogenes themselves (data not shown).

We should point out that high levels of H3K9me3 have also been observed in transcriptionally active genes, particularly at the 3′ exons of zinc finger genes [82], but this enrichment could be related to the presence of tandemly repeated domains and a potential role of H3K9me3 in preventing inappropriate recombination [83]. On this note, we noticed that our group II sRNA-producing pseudogenes showed slightly higher sequence similarity to their parents than group I pseudogenes, on average 90% *vs* 86% (p = 0.001, Wilcoxon test).

In addition to silencing pseudogenes, pseudogene-derived ncRNAs could be a good source of endogenous siRNAs that interfere with the expression of protein-coding genes. We uncovered evidence for this (Fig. 5), but this has been well addressed previously [24], [27], [84] and recently demonstrated for the human pseudogene*ψPPM1K* [30].

We were unable to address whether human pseudogene-derived sRNAs could function as siRNAs to interfere with parental gene expressions or to mediate epigenetic silencing under normal physiological conditions, since the necessary sRNA-Seq and histone modification ChIP-Seq data had not been available for all the 16 tissues in our current study. It will be interesting to revisit this critical issue when the relevant data become available for all or a subset of these tissues, so that data from the same tissues can be studied in order to reduce biological variations.

Our study indicates that the predominant effect of pseudogene transcription, however, appears to be related to the increase in the expression levels and diversity of the parental coding genes. Approximately 64% of the ∼4,000 transcribed pseudogenes (FPKM >1) exhibited a transcriptional profile that was positively correlated with that of their parental genes. Furthermore, pseudogenes with higher correlation were found to have more predicted miRNA-targeting sites. More importantly, the expression of parental genes was significantly higher and more variable in tissue where pseudogene ncRNAs are more abundant. All of these observations are consistent with previous reports suggesting that cellular RNAs could serve as miRNA sponges (or “target mimicry”) and regulate the stability of other transcripts [28], [85]–[88]. The most prominent case is *PTENP1*, whose transcripts have been shown by an extensive array of genetic and biochemical experiments to compete with its parental gene *PTEN* for several miRNAs [28]. The focus of our analysis is parental genes, but the implicated mechanism is applicable to the expression of all coding paralogs of a transcribed pseudogene. To fully decipher the intertwined interaction between pseudogene ncRNAs and other cellular RNAs, we need to extend our analysis using a network-based approach [89] and to simultaneously consider genes and pseudogenes in the same family [90] in the future. We should mention that 3′-UTRs are typically not annotated for pseudogenes because pseudogene detection is primarily based on aligning protein sequences to the human genome [91], but GENCODE manual annotation includes 3′-UTRs for some pseudogenes. Inclusion of additional 3′ sequences in the prediction of miRNA binding sites within pseudogenes may further improve our findings. Finally, our study only addresses the interaction at the transcription level and thus misses the potential importance of pseudogenes as miRNA decoys for regulating mRNA translation, though it has been shown that the majority of human miRNAs repress their targets by both reducing the level of mRNA transcripts and curtaining translation [92], [93].

In summary, consistent with previous work, our study demonstrates that pseudogene transcription is genuine and prevalent; its impact on other cellular RNAs appears complicated and diverse and in some cases one pseudogene may play multiple molecular roles. Whether these result passively from transcriptional leakage, incomplete chromatin silencing, or represent an active process of nurturing novel ncRNAs certainly will require a more systematic investigation and experimental verification. Our results, on the other hand, suggest that pseudogenes with major regulatory roles are unlikely ubiquitous, since under normal physiological conditions most of the pseudogene ncRNAs are present at much (∼20x) lower levels than their coding counterparts and the majority of them did not display evidence of strong interaction with their parents. Nevertheless, perturbation of pseudogene transcription can affect the homeostasis of gene expression and lead to human diseases and cancers as previously reported.

## Materials and Methods

### Data source

Our primary datasets are RNA-Seq data from the Illumina Body Map 2.0 Project (accession no. E-MTAB-513, http://www.ebi.ac.uk/arrayexpress), and transcriptomic and epigenomic data from the ENCODE project (http://genome.ucsc.edu/ENCODE/) [39]. The former is polyA+ selected mRNA sequencing data from 16 human tissues generated by Illumina Inc. for public usage; the paired-end reads were 50 or 75 bases long. A total of 3,775 million reads for all samples and 23.6 million reads on average per sample were analyzed. The specific ENCODE data used here are polyA+ RNA-Seq data as well as ChIP-Seq data of eight histone modifications (H3K27ac, H3K27me3, H3K36me3, H3K4me1, H3K4me2, H3Kme3, H3K9ac and H3K9me3) from the lymphoblastoid cell line GM12878. Sequencing data of small RNAs in two cell lines (GM12878 and K562) were also downloaded from the ENCODE project. A total of 36.6, 37.4, and 27.8 million reads for small RNAs derived from whole cell, cytosol, and nuclei of GM12878 cells, respectively, and correspondingly 12.7, 29.4, and 14.2 million reads for K562 cells were analyzed. We only present results based on analysis of combined small RNA reads, but separation of small RNAs by their cellular localizations yielded similar numbers. The genomic coordinates of integrated transcription factor binding sites (“wgEncodeRegTfbsClustered” track in the UCSC Table Browser) were also downloaded from the ENCODE project. Annotated lincRNAs and their corresponding expression values were obtained from the Human LincRNA Catalog described previously [41]. Pseudogenes were annotated manually and described by the GENCODE team in a recent report [11], including 8,716 processed pseudogenes, 2,158 duplicated pseudogenes, and 138 unitary pseudogenes. The original annotation contained 11,216 pseudogenes, but eleven of them overlapped with lincRNAs and therefore were excluded from the current study.

### Computational pipeline for screening RNA-Seq reads derived from pseudogenes

We preprocessed the RNA-Seq reads in order to reduce ambiguity in quantifying pseudogene transcription. This step is critical because the high sequence similarity shared by a pseudogene and its parental gene can lead to a great uncertainty in determining the *bona fide* origins of short RNA-Seq reads when both appeared to be equally good candidates. We first aligned all RNA-Seq reads to the human genome (hg19) and then collected reads mapped to pseudogene loci. These reads were then compared to cDNA sequences of human protein coding genes, extracted from the Ensembl database (http://www.ensembl.org/, Build 60), using the program Bowtie (version 0.12.7) [40]. Only reads mapped uniquely to a pseudogene (i.e., with fewer mismatches to the pseudogene than to any other cDNAs of annotated genes) were collected as “pseudogene reads”. Next, we edited the original RNA-Seq alignment files from Bowtie and deleted any entry associated to pseudogene loci if the reads were not in our list of pseudogene reads. Reads aligned to non-pseudogene loci were not edited. The modified alignment files were then used for our subsequent analysis of pseudogene transcription (Fig. 1A). To evaluate our approach's capability of resolving reads, we utilized the program T-coffee (version 9.03) [94] to align each pseudogene sequence to the corresponding coding exons of its parent, and then analyzed numbers of RNA-Seq reads *vs* sequence identities using a 200-bp window sliding across the alignment. Noted that all pseudogenes with an annotated parent (n = 9,459) were included in this analysis. According to pseudogene identification strategies, the aligned regions are essentially the “exons” of pseudogenes. A moderate correlation between read numbers and identities (Pearson correlation *r* = 0.202, *p*<2.2e-16; heart sample) was detected before the application of our filtering process, but none existed after filtering (*r* = 0.02, *p* = 0.48) (Fig. 1B, the same trend was observed in other tissue samples), indicating that RNA-Seq reads mapped to pseudogenes with our method were unlikely to have originated from parent genes.

The selected RNA-Seq reads for pseudogenes and reads mapped to the rest of the human genome were then used to compute expression values of all annotated transcripts by the program Cufflinks (version 0.9.3) [40]. Human transcript annotation was collected from the Ensembl database. Transcript abundances for each gene (or the combined gene expression) were calculated in Fragments Per Kilobase of exon per Million fragments mapped (FPKM). The expression values for the coding genes were hardly affected by read filtering (Fig. S1). The read filtering script, additional data, and other relevant scripts are available from the authors upon requested.

The read filtering step for computing FPKMs from RNA-seq data was not applied to either the ChIP-seq or sRNA-seq alignment data since the alignments of those reads were not be affected by exon-exon junction and a single and best-matched location was kept for each of the ChIP-seq reads.

### Measurement of transcriptional tissue specificity

JS (Jensen-Shannon) divergence has been found to be a good metric for quantifying the tissue specificity of a transcript [95]. We used the method described previously for computing JS scores of lincRNAs [41], which basically quantified the similarity between a transcript's expression across 16 tissues and a predefined extreme case in which the transcript was only present in one of the 16 tissues. As this computing resulted in 16 JS scores for each transcript, we picked the maximal JS score as in the previous study [41], whereas larger JS scores represent higher tissue specificity.

### Transcription correlation coefficient between pseudogenes and their parents

We computed Spearman correlation coefficient (ρ) of the 16 tissue FPKMs to determine the relationship between the transcription profiles of a pseudogene and its parent (ρ_pg:g_). As a control, we computed ρ by pairing up each pseudogene with a randomly chosen coding gene. Exclusion from this analysis were the pseudogenes without parent information from GENCODE or whose parents had 0 FPKMs in all the 16 tissues. The same method was applied to compute the correlation between miRNAs and their putative targets.

### Determination of transcriptional strands for pseudogenes

Strand-specific RNA-Seq data were obtained from a previous study [46]. The reads from all four samples (GEO: GSE32307) prepared from both ECC-1 (a human endometrial cancer cell line) and Universal Human Reference RNA library were combined and aligned to the human genome using Bowtie [40] and the same parameters as described [46]. After alignment data were filtered by our read-filtering pipeline, the number of reads within individual pseudogene loci was summed and normalized by the pseudogene length to yield expression values for both sense and antisense strands independently. Pseudogenes with a non-zero expression value were defined as sense (or antisense) transcription if the expression value for the annotated (or the opposite) strand was 10 times greater than the other strand. Those without a 10-fold distinction were considered as transcribed from both directions, which were likely overestimated. Among all pseudogenes with unique reads in the dataset (28% for duplicated and 55% for processed pseudogenes), the majority (393 and 66% for duplicated; 3122 and 65% for processed) exhibited evidence of sense transcription, whereas 134 duplicated and 891 processed pseudogenes were determined to produce ncRNAs from the antisense strand. Application of the same rules to coding genes resulted in an estimated error rate of <14% when the predicted strands were compared to the annotated ones, although a slight increase of FPKM cutoff (0.05) would result in a much smaller error rate (<5%).

### miRNA target prediction

We downloaded data of miRNA sequence families and target prediction tools (TargetScan V5.0) from the TargetScan web site (http://www.targetscan.org) [96]. Only the “exonic” sequences of pseudogenes (or genes) were used for predicting miRNA target sites with default parameters of TargetScan. We also analyzed the miRNA-mRNA interactions that were experimentally determined by the CLASH analysis [49].

### Analysis of pseudogenes producing small RNAs

Using the sRNA-seq alignment data for GM12878 and K562 cells, we counted the numbers of sRNA reads within genes or pseudogenes, and then normalized the counts by gene or pseudogene lengths to obtain sRNA read densities, as sRNA reads per kb. The pseudogenes with ≥5 sRNA reads per kb in their “exonic” regions (1,549 for GM12878 and 2,092 for K562; p<0.001, Poisson test) were considered as candidates that could produce small RNAs. A subset of these candidates were defined as group I or II sRNA-generating pseudogenes if the sRNA read densities at their flanking 1 kb regions were  = 0 or >5, respectively (Fig. 5; Table S1); the rest were not analyzed further. The rationale behind this separation is that pseudogene-derived sRNAs may have two kinds of very distinct functions: one is to interact and interfere with the expression of parental genes by the small RNA inference mechanism while the other is to recruit chromatin modifiers to repress pseudogenes in a manner similar to repeat/transposon-derived sRNAs for heterochromatin formation. For the former (i.e., group I), sequence complementary between mRNAs and pseudogene ncRNAs is required and the detection of sRNAs in both parents and pseudogenes but not in their flanking genomic regions is expected, though the sRNA biogenesis may arise from hairpin loops in pseudogene ncRNAs alone or between pseudogene ncRNAs and other complementary transcripts [22], [23], [30]. For the latter (i.e., group II), it is unnecessary to observe sRNAs in the parents, but technically it would be difficult for us to exclude them as potential origins of pseudogene-derived siRNAs. Therefore, the assignment of group II is based on the hypothesis that sRNAs would also be likely to originate from pseudogene flanking regions, which are not shared between pseudogenes and their parents, because siRNA-mediated epigenetic silencing is often extended to a relatively large chromatin region as shown in plants, yeast and flies [50], [51], [53].

### Characterization of histone modifications at pseudogenes

In the comparison of transcribed *vs* non-transcribed pseudogenes, we evaluate the difference by considering the number of mapped ChIP-Seq reads at ±2.5kb of pseudogene TSS (Fig. S6). The significant difference was detected using either transcribed pseudogenes from all tissues or only those from GM12878 and K562 cells. In the analysis of the relationship between sRNAs and H3K27me3 and H3K9me3, we considered ChIP-Seq reads at pseudogene bodies. The patterns in Figure S6 did not change significantly when pseudogenes TSSs overlapping with coding exons or gene TSSs were excluded from the analysis.

### Analysis of pseudogene conservation

Sequence conservation was determined by the Phastcon scores downloaded from the UCSC genome browser; the scores were derived from sequence comparison of 46 species including primates, mammals and vertebrates [97]. The conservation score for a pseudogene was measured as the mean of phastcon scores for all base pairs within its “exons.” Nucleotide diversity for each pseudogene locus was derived using the formula below with SNP data for 161 and 160 individuals from the Yoruba (YRI) and European (CEU) population in the International HapMap project (http://www.hapmap.org) [98], respectively.
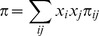
where 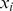
and 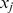 are the respective frequencies of the *i*-th and *j*-th individual sequences from YRI population, 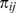 is the number of nucleotide differences per nucleotide site between the *i*-th and *j*-th individual sequences in each pseudogene locus [99]. Pseudogenes with 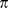 = 0 in either YRI or CEU were excluded from our comparison of transcribed vs non-transcribed pseudogenes, in order to avoid the potential complication that some recently emerging pseudogenes have not been fixed in human population. The data shown in Figure 7 were derived from combined analysis of YRI and CEU data.

## Supporting Information

Figure S1 **Most pseudogenes share <90% sequence similarity with their parents and our method for filtering RNA-Seq reads does not affect the quantification of parental gene expression.** Left, histogram of human pseudogene distribution shows the number of pseudogenes (y-axis) at different levels of sequence identity to the parental genes (x-axis). Right, the FPKM values for the parental genes is not affected by our method of filtering and remapping of RNA-Seq reads. Data shown is for brain sample, but results from other tissues yielded the same pattern.
(PDF)

Figure S2 **Tissue specificity of pseudogene transcription.** A) Three examples of tissue-restrictively transcribed pseudogenes. B) Distribution of the JS scores computed with all RNA-Seq reads (yellow, also in Figure 2B) for lincRNAs, pseudogenes, and genes is very similar to that derived with ½ of the total RNA-Seq reads (blue). To generate one half of the data, we randomly picked one of the two replicates for each tissue. (C). Distribution of JS scores computed for pseudogenes and randomly selected genes with matching maximal FPKMs in the 16 tissues.
(PDF)

Figure S3 **(A–B) QQ-plot analysis of transcriptional correlation coefficients.** The ρ_pg:g_ values (y-axis) for transcribed duplicated pseudogenes (A) and processed pseudogenes (B) were significantly deviated from the ρ_pg:g_ values (x-axis) calculated for pairs of each transcribed pseudogenes with a randomly chosen coding genes. C) Distinct effect on parental gene expression between sense and antisense pseudogene ncRNAs. The parents of the antisense transcribed pseudogenes (n = 382, green) exhibited significantly lower expression than those of sense transcribed pseudogenes (n = 1538, red) in all the 16 tissues (p<0.05, Wilcoxon test).
(PDF)

Figure S4 **Increased levels and variations of parental gene expression in relation to pseudogene transcription.** QQ-plot analysis shows that both differences in mean (A) and variance (B) of the parental gene expression between tissues of high (μ_h_, *S_h_*) and low (μ_l_, *S_l_*) pseudogene transcription were significantly deviated from the normal distribution; the Kolmogorov-Smirnov (KS) statistics are shown at top. The means (C) and variances (D) of the expression of the parent genes (x-axis) across all 16 human tissues also increased as the transcription levels of pseudogenes increased. Color lines plot the distributions of parental genes with pseudogenes transcribed at different levels, defined by the maximal FPKMs among the 16 tissues.
(PDF)

Figure S5 **Size distribution of small RNAs from the group I and II pseudogenes.** The data were derived from the sRNA reads that were perfectly matched to pseudogene sequences without any gap. Data from GM12878 and K562 are plotted in (A; p<1e-05) and (B; p<0.0002), respectively.
(PDF)

Figure S6 **Enrichment of active histone modifications and depletion of repressive histone modifications at transcribed pseudogene loci.** Comparison of eight histone modifications between transcribed (‘y’) and non-transcribed (‘n’) pseudogenes was shown by boxplot analysis. The y-axis shows numbers of ChIP-Seq reads mapped to +/− 2.5 kb to TSS in GM12878 (A) and K562 (B) cell lines. C). The average densities of three types of repeats at pseudogenes transcribed in GM12878 (FPKM>1) in 500-bp bin windows, with no enrichment observed at pseudogene loci when compared to adjacent genomic regions.
(PDF)

Figure S7 **Correlation of the nucleotide diversities computed from two distinct human populations.** For every pseudogene, we determined its nucleotide diversities in the YRI or CEU populations and the results show a high correlation between the data derived from these two populations, indicating the reduction in diversity is not due to a few genes that recently become pseudogenes in human. A), duplicated pseudogenes; B), processed pseudogenes.
(PDF)

Table S1 **List of transcribed pseudogenes and their associated features and groups.**
(XLSX)
