# Supplementary figures and images for: Characterization of Human Pseudogene-Derived Non-Coding RNAs for Functional Potential
Source: PLoS One. 2014 Apr 3;9(4):e93972. doi: 10.1371/journal.pone.0093972 (PMC3974860; doi:10.1371/journal.pone.0093972)

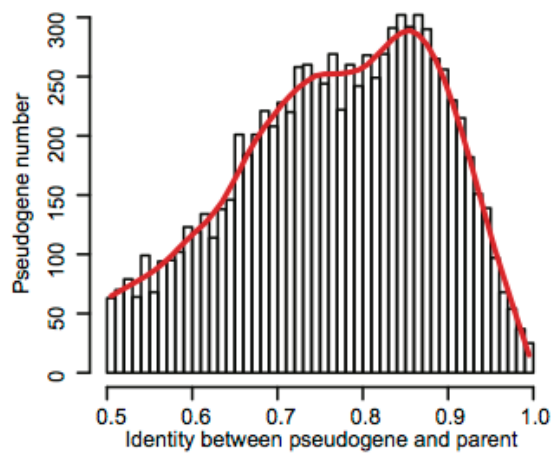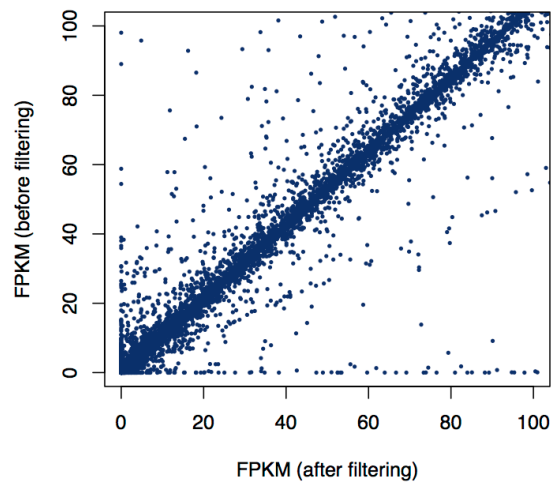

Supplement: Figure S1 — Most pseudogenes share <90% sequence similarity with their parents and our method for filtering RNA-Seq reads does not affect the quantification of parental gene expression. Left, histogram of human pseudogene distribution shows the number of pseudogenes (y-axis) at different levels of sequence identity to the parental genes (x-axis). Right, the FPKM values for the parental genes is not affected by our method of filtering and remapping of RNA-Seq reads. Data shown is for brain sample, but results from other tissues yielded the same pattern. (PDF) [file pone.0093972.s001.pdf]

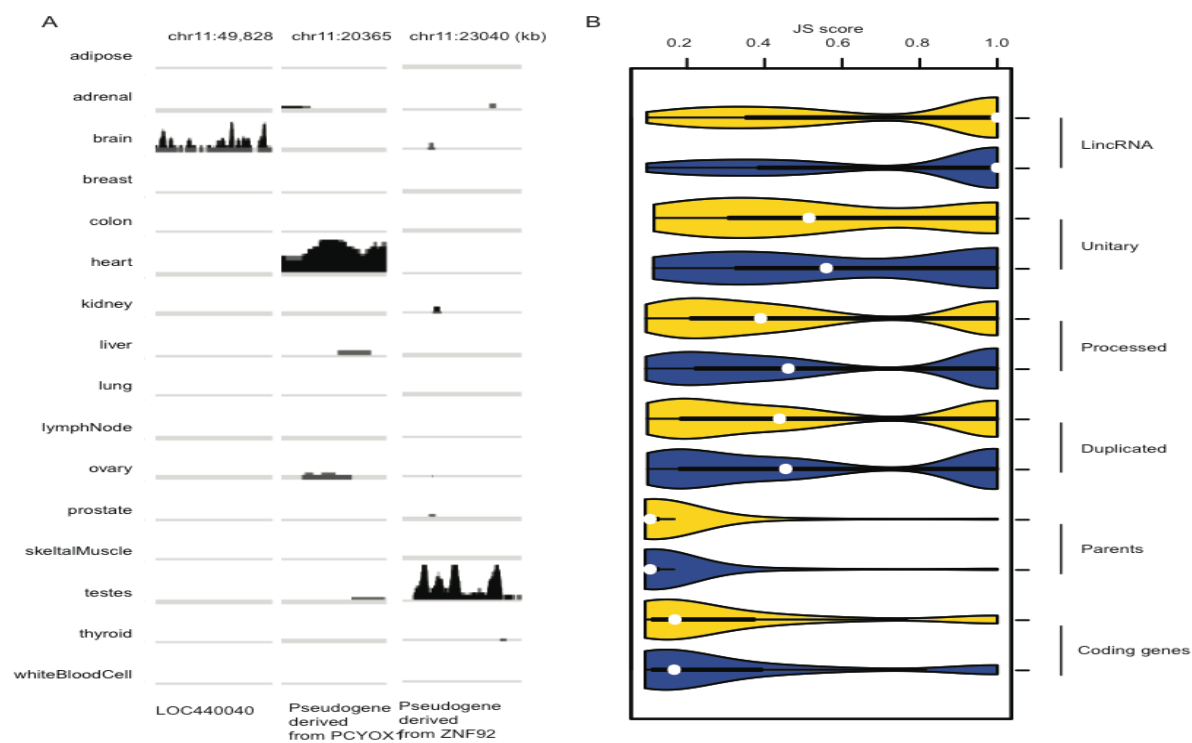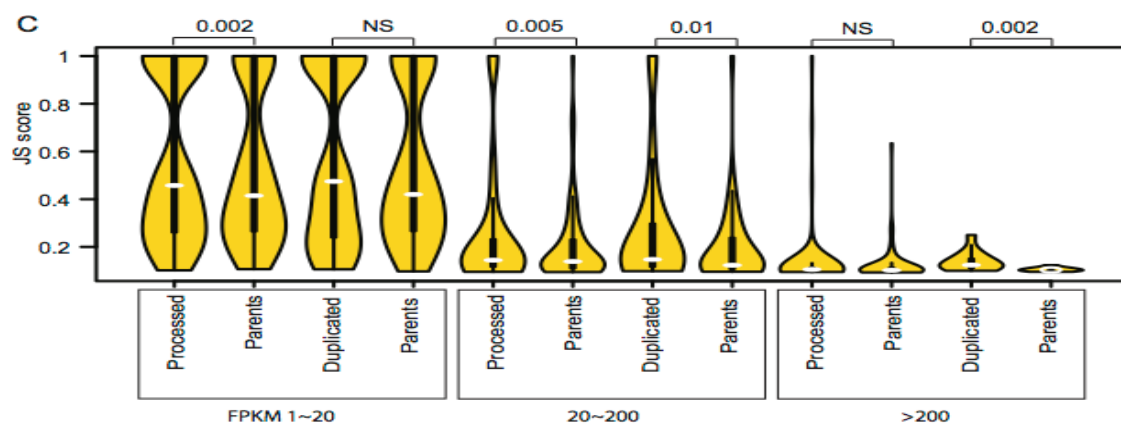

Supplement: Figure S2 — Tissue specificity of pseudogene transcription. A) Three examples of tissue-restrictively transcribed pseudogenes. B) Distribution of the JS scores computed with all RNA-Seq reads (yellow, also in Figure 2B) for lincRNAs, pseudogenes, and genes is very similar to that derived with ½ of the total RNA-Seq reads (blue). To generate one half of the data, we randomly picked one of the two replicates for each tissue. (C). Distribution of JS scores computed for pseudogenes and randomly selected genes with matching maximal FPKMs in the 16 tissues. (PDF) [file pone.0093972.s002.pdf]

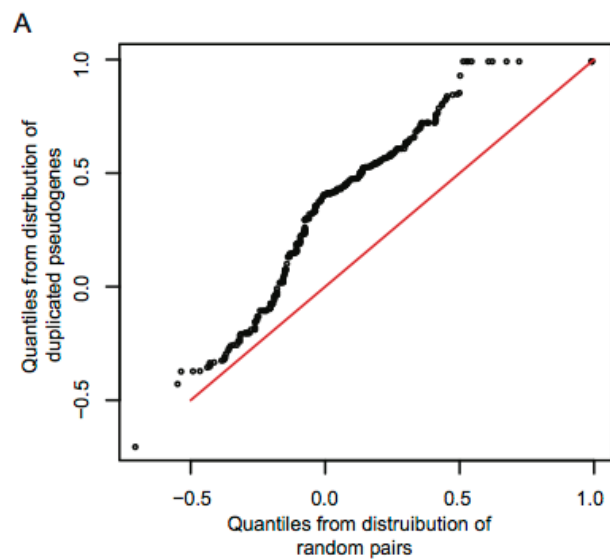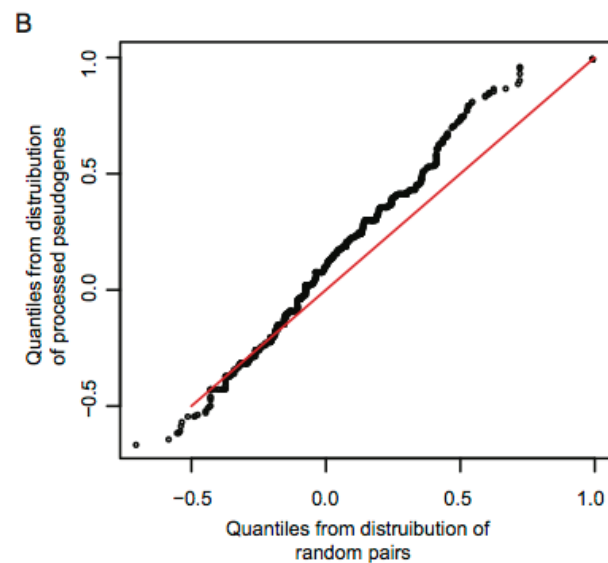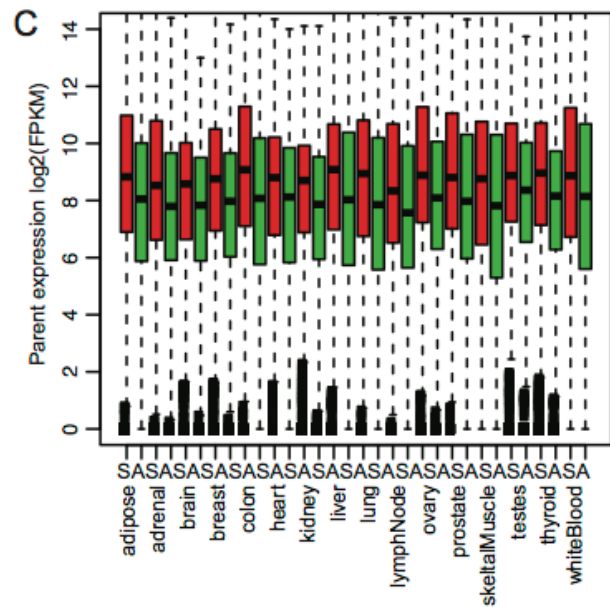

Supplement: Figure S3 — (A–B) QQ-plot analysis of transcriptional correlation coefficients. The ρpg:g values (y-axis) for transcribed duplicated pseudogenes (A) and processed pseudogenes (B) were significantly deviated from the ρpg:g values (x-axis) calculated for pairs of each transcribed pseudogenes with a randomly chosen coding genes. C) Distinct effect on parental gene expression between sense and antisense pseudogene ncRNAs. The parents of the antisense transcribed pseudogenes (n = 382, green) exhibited significantly lower expression than those of sense transcribed pseudogenes (n = 1538, red) in all the 16 tissues (p<0.05, Wilcoxon test). (PDF) [file pone.0093972.s003.pdf]

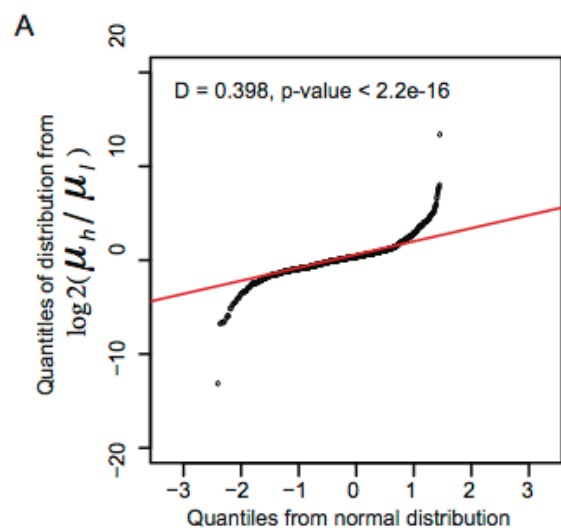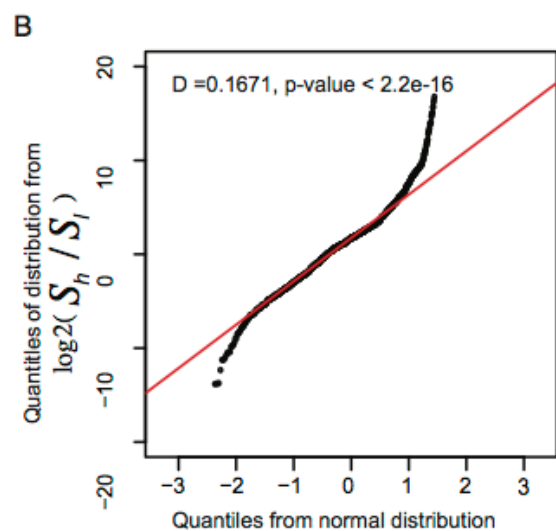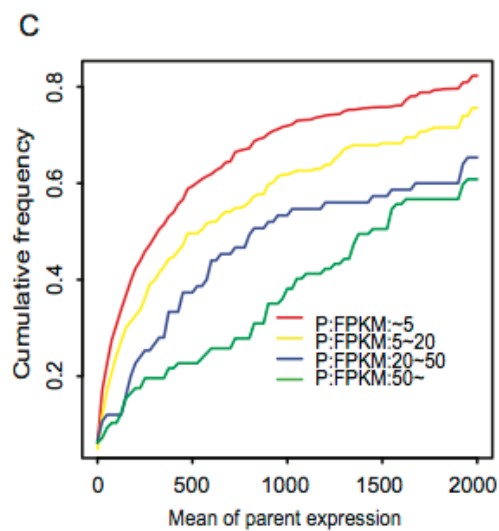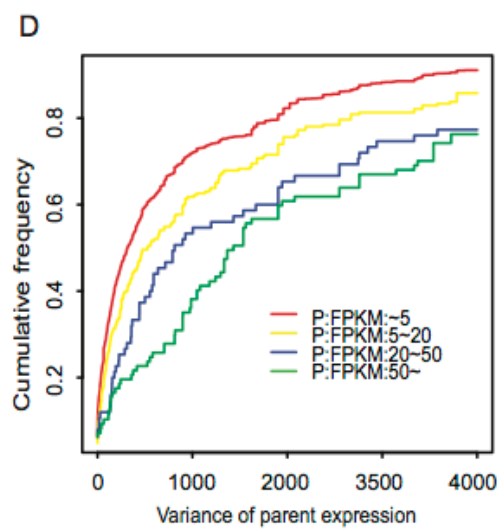

Supplement: Figure S4 — Increased levels and variations of parental gene expression in relation to pseudogene transcription. QQ-plot analysis shows that both differences in mean (A) and variance (B) of the parental gene expression between tissues of high (μh, Sh) and low (μl, Sl) pseudogene transcription were significantly deviated from the normal distribution; the Kolmogorov-Smirnov (KS) statistics are shown at top. The means (C) and variances (D) of the expression of the parent genes (x-axis) across all 16 human tissues also increased as the transcription levels of pseudogenes increased. Color lines plot the distributions of parental genes with pseudogenes transcribed at different levels, defined by the maximal FPKMs among the 16 tissues. (PDF) [file pone.0093972.s004.pdf]

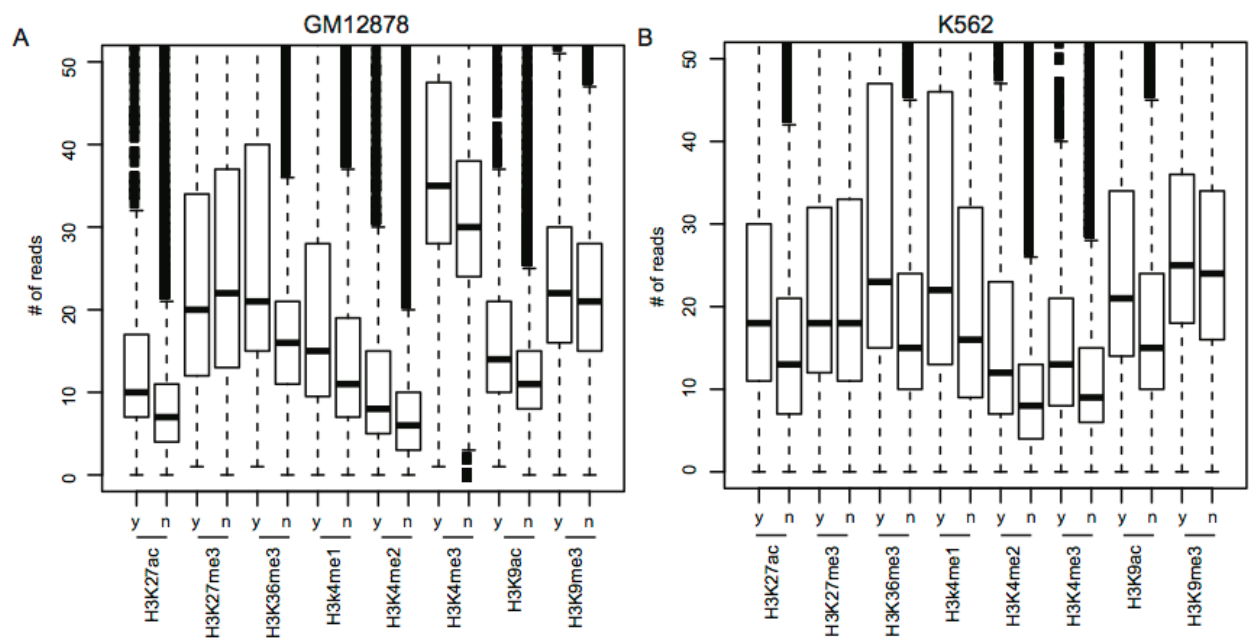

**C**

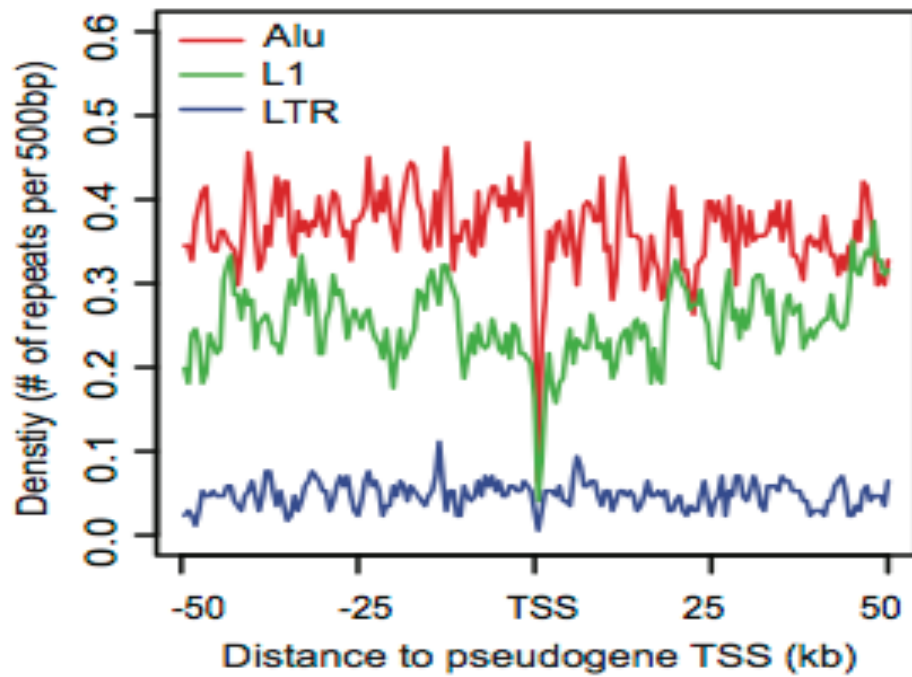

Supplement: Figure S6 — Enrichment of active histone modifications and depletion of repressive histone modifications at transcribed pseudogene loci. Comparison of eight histone modifications between transcribed (‘y’) and non-transcribed (‘n’) pseudogenes was shown by boxplot analysis. The y-axis shows numbers of ChIP-Seq reads mapped to +/− 2.5 kb to TSS in GM12878 (A) and K562 (B) cell lines. C). The average densities of three types of repeats at pseudogenes transcribed in GM12878 (FPKM>1) in 500-bp bin windows, with no enrichment observed at pseudogene loci when compared to adjacent genomic regions. (PDF) [file pone.0093972.s006.pdf]

**A**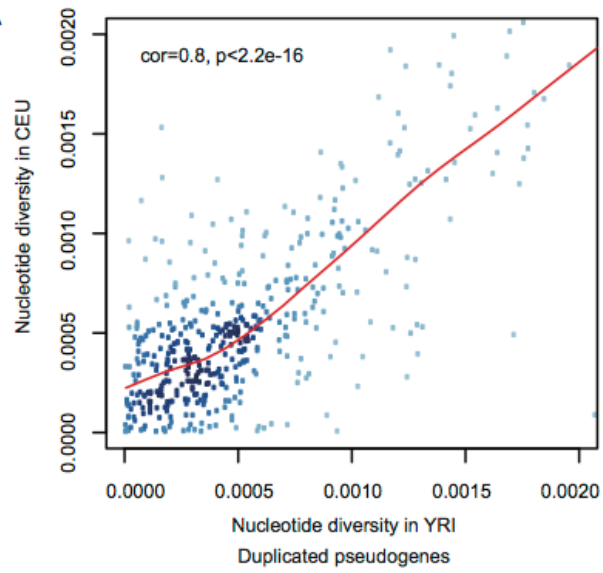**B**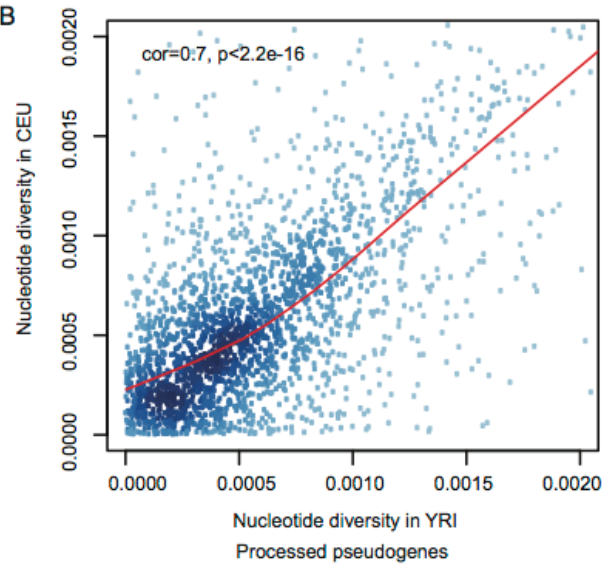

Supplement: Figure S7 — Correlation of the nucleotide diversities computed from two distinct human populations. For every pseudogene, we determined its nucleotide diversities in the YRI or CEU populations and the results show a high correlation between the data derived from these two populations, indicating the reduction in diversity is not due to a few genes that recently become pseudogenes in human. A), duplicated pseudogenes; B), processed pseudogenes. (PDF) [file pone.0093972.s007.pdf]
