# Supplementary material for: Characterization of Human Pseudogene-Derived Non-Coding RNAs for Functional Potential
Source: PLoS One. 2014 Apr 3;9(4):e93972. doi: 10.1371/journal.pone.0093972 (PMC3974860; doi:10.1371/journal.pone.0093972)
Supplement: Figure S5 — Size distribution of small RNAs from the group I and II pseudogenes. The data were derived from the sRNA reads that were perfectly matched to pseudogene sequences without any gap. Data from GM12878 and K562 are plotted in (A; p<1e-05) and (B; p<0.0002), respectively. (PDF) [file pone.0093972.s005.pdf]

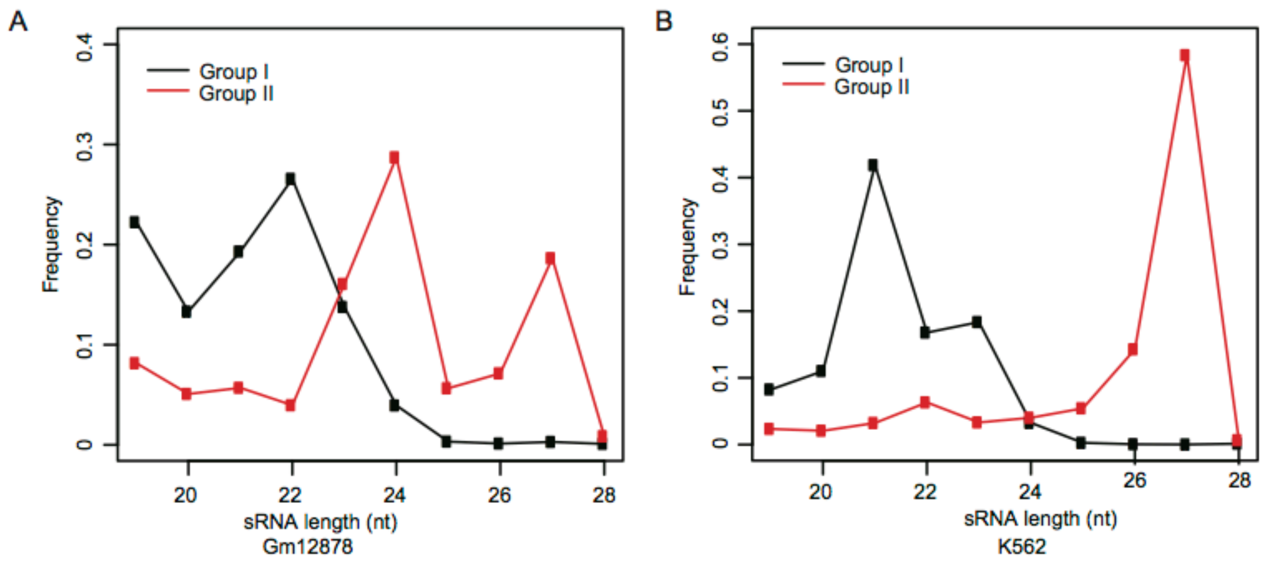

** These distributions were derived from a total of 3,071 and 101,391 mapped sRNA reads for I and II in GM12878, respectively. The total for K562 is 5,971 and 123,364.
